# Calculating the serial interval of SARS-CoV-2 in Lebanon using 2020 contact-tracing data

**DOI:** 10.1186/s12879-021-06761-w

**Published:** 2021-10-11

**Authors:** Nadine Haddad, Hannah Eleanor Clapham, Hala Abou Naja, Majd Saleh, Zeina Farah, Nada Ghosn, Pamela Mrad, Natasha Howard

**Affiliations:** 1 Epidemiological Surveillance Programme, Ministry of Public Health, Beirut, Lebanon; 2 Cyprus International Institute for Water and Health Laboratory, Cyprus University of Technology, Limassol, Cyprus; 3 Saw Swee Hock School of Public Health, National University of Singapore, 12 Science Drive 2, Singapore, 117549 Singapore; 4 Department of Global Health and Development, London School of Hygiene and Tropical Medicine, 15-17 Tavistock Place, London, WC1H 9SH UK; 5 Lebanon Country Office, World Health Organization, Beirut, Lebanon

**Keywords:** SARS-CoV-2, Contact tracing, Serial interval, COVID-19

## Abstract

**Introduction:**

The first detected case in Lebanon on 21 February 2020 engendered implementation of a nationwide lockdown alongside timely contact-tracing and testing.

**Objectives:**

Our study aims to calculate the serial interval of SARS-CoV-2 using contact tracing data collected 21 February to 30 June 2020 in Lebanon to guide testing strategies.

**Methods:**

rRT-PCR positive COVID-19 cases reported to the Ministry of Public Health Epidemiological Surveillance Program (ESU-MOH) are rapidly investigated and identified contacts tested. Positive cases and contacts assigned into chains of transmission during the study time-period were verified to identify those symptomatic, with non-missing date-of-onset and reported source of exposure. Selected cases were classified in infector–infectee pairs. We calculated mean and standard deviation for the serial interval and best distribution fit using AIC criterion.

**Results:**

Of a total 1788 positive cases reported, we included 103 pairs belonging to 24 chains of transmissions. Most cases were Lebanese (98%) and male (63%). All infectees acquired infection locally. Mean serial interval was 5.24 days, with a standard deviation of 3.96 and a range of − 4 to 16 days. Normal distribution was an acceptable fit for our non-truncated data.

**Conclusion:**

Timely investigation and social restriction measures limited recall and reporting biases. Pre-symptomatic transmission up to 4 days prior to symptoms onset was documented among close contacts. Our SI estimates, in line with international literature, provided crucial information that fed into national contact tracing measures. Our study, demonstrating the value of contact-tracing data for evidence-based response planning, can help inform national responses in other countries.

**Supplementary Information:**

The online version contains supplementary material available at 10.1186/s12879-021-06761-w.

## Introduction

Since its initial designation as a public health event of international concern on January 30, 2020 and as a pandemic on March 11, 2020 by the World Health Organization (WHO) [1], COVID-19—the disease caused by severe acute respiratory syndrome coronavirus 2 (SARS-CoV-2)—pushed countries worldwide to implement a range of public health and social measures to limit virus spread and break transmission chains. Such measures, relying mainly on physical distancing, ranged from partial or full closures of retail, recreational, educational, work, and religious institutions and implementation of international or domestic travel restrictions. By the end of March 2020, over a hundred countries worldwide were on partial or full lockdown [2]. To improve understanding of COVID-19 epidemiological, clinical, and virological characteristics, WHO initiated the First Few X cases and contacts (FFX) investigation protocol, aiming to identify transmission dynamics, severity, and clinical spectrum. FFX allows description of clinical presentation and severity, as well as estimation of serial interval, secondary infection rate (SIR), and the proportion of symptomatic cases [3].

In Lebanon, the first COVID-19 case was detected on February 21 2020 in a symptomatic passenger returning on a flight from Iran. Subsequently, passengers returning from countries with clusters or sustained local transmission were screened for symptoms at the airport and land crossings and followed-up daily for the next 14 days. Travelers developing symptoms were referred for testing using real-time Reverse Transcription Polymerase Chain Reaction (rRT-PCR) at Rafik Hariri University Hospital (RHUH). Decisions on public health and social measures rapidly accelerated thereafter, with nurseries, schools and universities closed between February 29 and March 2, followed by closures of restaurants, cafés and night clubs on March 10 when the first confirmed COVID-19 death was declared. On March 11, as COVID-19 was declared a pandemic by WHO, Lebanon banned travel from 11 countries with local transmission. On March 15, the Council of Ministers and the Supreme Council of Defence declared a state of public health emergency imposing nationwide lockdown including closures of all ports of entry from March 16 [4]. On April 9, the national lockdown was extended until June 7. Lockdown measures were released in phases with the fifth and final phase targeting retail and recreational centres, which were given official permission to reopen on June 8 (with reduced capacity) and international flights, which resumed on July 1 (with 10% operational capacity compared to 2019) [5].

In the context of novel infectious pathogens, understanding transmission dynamics and parameters is crucial to better implementation of control and mitigation strategies. Examples of such parameters include the incubation period (i.e. time between exposure and onset of symptoms), generation time (i.e. time between the point when the infector is infected and then infects someone), serial interval (i.e. time between symptoms onset of an infector and that of an infectee), and reproduction number (i.e. average number of infections generated by an infectious individual) [6]. These parameters are critical for improving implementation of case isolation, contact tracing, and quarantine periods. The aim of this study was to estimate the serial interval of SARS-CoV-2 in Lebanon using surveillance data collected from the first few hundred cases and contacts in Lebanon as per the WHO FFX investigation protocol.

## Methods

### Case definitions

We used the COVID-19 case definition provided by Lebanese Ministry of Public Health (MOPH) circular no. 35, issued February 24, 2020, as ‘any person with any laboratory confirmation, including positive serology in paired serum samples, specific Polymerase Chain Reaction (PCR), or genome sequencing, irrespective of clinical signs and symptoms’ [7]. We also adopted the WHO case definition for close contact, as ‘any person who had direct physical or a close contact (within 1 m for more than 15 min) with a confirmed COVID-19 case; or any person providing direct care for patients with COVID-19 disease without the proper use of personal protective equipment (PPE), from 2 days before to 14 days after the case’s onset of symptoms [8].

### Data management

All private and public testing laboratories are requested to report confirmed COVID-19 cases daily to the Epidemiological Surveillance Program at the Lebanese Ministry of Public Health (ESU-MOH). National data entry, initially performed by ESU-MOH central office using the electronic DHIS2 platform, is performed by testing laboratories since September 2020. All confirmed cases were initially investigated using the FFX protocol “Case Initial Reporting Form A1” subsequently summarised as a two-page case investigation form issued by ESU-MOH, collecting similar information about socio-demographics, clinical signs and symptoms, contacts, and exposures in the 14 days prior to symptom onset. During the first few weeks of the national epidemic, all confirmed cases were isolated at Rafik Hariri University Hospital (RHUH) in Beirut, until they tested negative twice using rRT-PCR. On March 30, an MOPH decree specified the duration of institutional or home isolation as 30 days from symptom onset for symptomatic cases and from date of confirmation for asymptomatic cases (MOPH decree 359/1 dated March 30, 2020) [9]. On November 6, MOPH circular no. 159 reduced self-isolation duration to 10 days for asymptomatic and 13 days for symptomatic cases in line with WHO guidance.

ESU-MOH investigated identified close contacts, using FFX protocol Form B1 (Contact initial reporting form) to collect socio-demographic information, exposure history and symptoms onset, and followed them for 14 days from last encounter with the confirmed case using Form B2 (Contact follow-up reporting form). Initially, all contacts were requested to home quarantine for 14 days and were tested on becoming symptomatic. From April 21, MOPH circular no. 73 requested all contacts be tested for COVID-19 once identified, regardless of symptoms. All symptomatic and asymptomatic cases and contacts testing positive—and subsequently classified as cases—were assigned to chains of transmission by ESU-MOH epidemiologists. Thus, the same chain of transmission can include infections acquired from household, occupational (i.e. work settings other than hospitals) and healthcare exposure (i.e. between healthcare workers, or between patients and healthcare workers).

### Constructing transmission pairs and calculating serial interval

For this study, we reviewed all cases reported to ESU-MOH DHIS2 COVID-19 central database between 21 February 2020 (i.e. detection of first case) and 30 June 2020 (i.e. cluster transmission phase) and retained those meeting all the following criteria: (i) reported at least one symptom (i.e. fever, cough, dyspnea, headache, myalgia/arthralgia, anosmia); (ii) provided a date of onset; and (iii) were linked to chains of transmission (i.e. non sporadic cases). Cases were excluded if they: (i) were asymptomatic (no reported symptoms); (ii) were symptomatic but did not provide a date of onset; or (iii) their exposure was multi-factorial including several primary cases or exposures. We reviewed transmission chains of retained cases by chronological order of exposure and symptoms onset to construct infector–infectee pairs as described by Xu et al. [10]. The ‘infector’ was defined as the primary case, with an identified source of exposure occurring prior to their encounter with the ‘infectee’ and presenting any symptoms. The ‘infectee’ was defined as the secondary case whose exposure was solely by the infector and presenting any symptoms.

In the same chain of transmission, the same infector could generate more than one pair if they infected more than one individual. Additionally, the infectee from one pair could become an infector for another pair. Thus, to be conservative, pairs were discarded if the same onset date was reported by both the infector and infectee, as this was suggestive of an external unidentified source of infection. For every pair, the time difference between the date of symptom onset of the infectee and that of the infector was calculated as number of days. To estimate the serial interval, we used the likelihood-based estimation method proposed by Wallinga and Teunis [11]. Confidence intervals were generated with bootstrapping of 1000 iterations. Analysis was conducted using R version 4.0.3 and R studio version 1.2.5033 and distribution fitting using **fitdistrplus** package.

## Results

Between February 21 and June 30, 2020, a cumulative number of 1788 confirmed COVID-19 cases were reported in Lebanon, which we screened for verification of source of exposure and onset date. We excluded 805 asymptomatic cases, 302 symptomatic cases with missing date of onset, and 532 cases with multi-factorial exposure or multiple possible primary cases. Table 1 shows a total of 149 cases (62 infectors, 87 infectees) included. We obtained 103 infector–infectee pairs from 24 chains of transmission as per our selection criteria. Seventeen individuals were simultaneously considered as an infector in one pair and an infectee in another.

**Table 1:** Distribution of cases as infector–infectee pairs

Number of pairs assigned to each infector	Number of infector (primary cases)	Number of generated infectees
1	35	35
2	15	30
3	8	24
4	1	4
5	2	10
Total	62	103

Table 2 shows no significant differences (p = 0.984) in the mean ages of infectors (50 ± 18.5 years) and infectees (45.3 ± 18.6 years). Most cases were Lebanese (98%) and male (63%). All infectees acquired the infection locally, as compared to 89% (n = 55) among infectors (p = 0.009).

**Table 2:** Participant characteristics

Demographics	Infector, n = 61 (%)	Infectee, n = 87 (%)	p-value
Age (mean years)	50 ± 18.5	45.3 ± 18.6	0.129
Sex			
Male	39 (63)	41 (46)	0.061
Female	23 (37)	48 (54)	
Nationality			
Lebanese	61 (98)	85 (98)	0.999
Syrian	1 (2)	2 (2)	
Source of infection			
Locally acquired	55 (89)	87 (100)	0.009
Imported	7 (11)	0 (0)	

For calculation of the serial interval from non-truncated data, all 103 identified pairs were included in analysis. The empirical density distribution plot of the serial interval of non-truncated data is shown in Additional file 1. The mean serial interval fitted to the normal distribution was 5.24 days (95% CI 4.37–6.03), with a minimum of − 4 and a maximum of 16 days (Table 3). Skewness and kurtosis were calculated at 0.56 and 3.02, respectively. The Shapiro–Wilk normality test was calculated at W = 0.962 (p-value = 0.005), implying the serial interval was merely normally distributed. Using maximum-likelihood estimation, the Akaike information criterion (AIC) of the fitted non-truncated data to the normal distribution is calculated as 578.76.

**Table 3:** Summary statistics of fitted distributions for non-truncated and truncated data

Data	Distribution model	Mean (95% CI)	AIC
Non-truncated	Normal (Mean, SD)	5.24 (4.37–6.03)	578.76
Truncated (> 0)	Normal (Mean, SD)	5.68 (4.94–6.46)	528.99
	Log-normal (meanlog = 1.51, sdlog = 0.70)	6.02 (5.06–6.97)	504.83
	Gamma Shape = 2.44, rate = 0.43	5.88 (5.65–6.11)	501.56
	Weibull Shape = 1.65, Scale = 6.36	5.78 (5.56–6.01)	503.24

NB: AIC is Akaike information criterion

Next, data analysis was repeated on truncated data after removing the six pairs with non-positive values of the serial interval. For the remaining 97 pairs, the mean serial interval fitted to the normal distribution was calculated at 5.68 days (95% CI 4.94–6.46). Skewness and kurtosis were calculated at 0.85 and 2.93, respectively. The Shapiro–Wilk normality test was calculated at W = 0.911 (p-value = 1.01 × 10^5^). The Akaike information criterion (AIC) of the fitted truncated data to the normal distribution is calculated as 528.99.

**Fig. 1:**
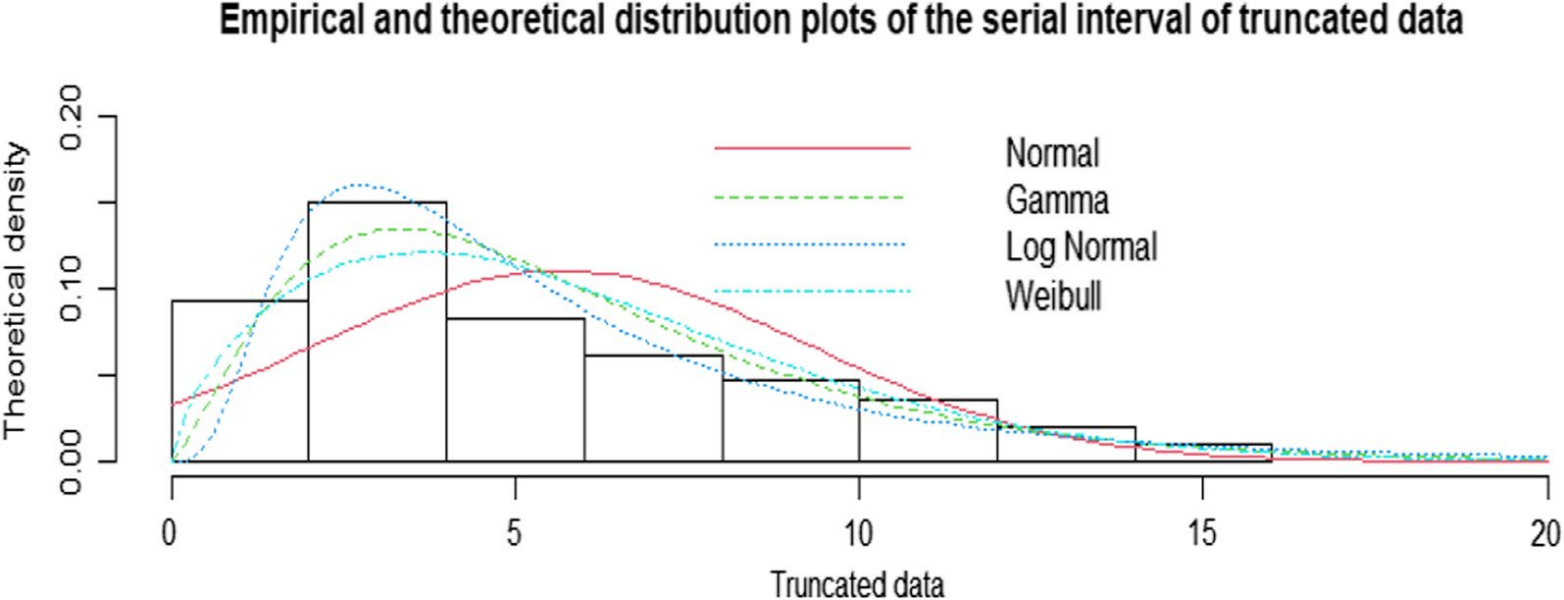
Empirical and theoretical distribution plots of the serial interval of truncated data

Three other distributions were fitted to the non-truncated data and their AIC evaluated: normal, log-normal, Gamma and Weibull. Fitting the log-normal distribution to the non-truncated data, the fitted mean serial interval was estimated as 6.02 days (95% CI 5.06–6.97), AIC = 504.83. The fitted mean was estimated at 5.88 (95% CI 5.65–6.11), AIC = 501.56 from the Gamma distribution and at 5.78 (95% CI 5.56–6.01), AIC = 503.24 for Weibull distribution. The empirical and theoretical density distribution plots of the serial interval of non-truncated data are shown in Fig. 1.

## Discussion

The serial interval estimates presented were computed using the first COVID-19 case data from Lebanon’s epidemic as reported to the national surveillance program. Our findings revealed estimates of 5.24 days (95% CI 4.37–6.03) for complete data, and at 5.68 days (95% CI 4.94–6.46) for truncated data between February 21 and June 30, 2020. Findings from both complete and truncated data are in line with those found in the literature. A study conducted in China, outside Hubei province, estimated the mean SI 5.2 (± 4.7) days when rapid case identification and isolation measures were implemented, comparable to our SI estimates when similar COVID-19 surveillance and response activities were implemented [12]. Another study in mainland China, covering January–February 2020, estimated SI as 5 (95% credible interval [CrI] 4.4–5.5) and 5.2 (95% CrI 4.9–5.7) days for household and non-household transmission respectively [10]. Further, a systematic review of 23 studies conducted between February and March 2020 estimated the mean SI between 4.2 to 7.5 days, with a pooled weighted mean estimate of 5.2 (95% CI 4.9–5.5) days [13]. Similarly, a systematic review of 11 studies conducted during the same period found a pooled estimate for SI as 5.40 (5.19, 5.61) and 5.19 (4.37, 6.02) days by the fixed and random effects model, respectively [14]. However, a study in Japan, using publicly-available data at the start of the pandemic, found the mean SI to be 4.7 (95% CrI 3.7, 6.0) days, which was slightly lower than reported estimates at the start of the epidemic in China possibly due to the small sample size limited to only 28 studied pairs of infector–infectees [15].

Though both complete and truncated data are reported in the literature, we do not think there is a valid reason to exclude negative values from the data as this might exclude valuable information. Therefore, looking at our complete non-truncated data, we found that the normal distribution provides the best fit, which has also been used in other studies [10, 11, 14]. As documented elsewhere, we consider that the normal distribution can be used with confidence for future epidemiological assessment and modelling [10].

Interestingly, looking at our non-truncated data, a pre-symptomatic transmission (i.e. where the infectee developed symptoms before the infector-primary case) was documented from 4 days before symptoms onset. Examination of the six pairs with negative serial interval values reveals these occurred in the same household, suggesting that pre-symptomatic transmission can occur among close contacts [10, 12]. Our results align with those of a study of 468 confirmed cases in China reported in February 2020 [16], and with the earliest recommendations of the WHO-FFX protocol that defines a contact as someone who was in contact with a positive case from 1–4 days before symptom onset [3]. Nevertheless, the contact tracing procedures followed by MOPH Lebanon were built upon WHO considerations, defining contacts from 2 days before to 14 days after the case’s onset of illness [8].

The main strength of our study resides in the thorough contact tracing undertaken from detection of the first case in the country. The proportion of travel-acquired infections in our study sample was significantly higher among infectors (11.3%) as compared to infectee, in line with surveillance findings at national level during the same time-period, when community transmission was not yet established in the country. The study period, which corresponded with a country-wide lockdown and limited cluster transmission, minimized risk of reporting bias for possible sources of infection as was reflected by Nishiura et al. [16]. Additionally, the timely investigation of both cases and contacts reduced possible recall bias of the dates of symptoms onset, which was reported as a possible limitation in other studies [11].

This study is limited to estimating SI parameters in symptomatic cases. It would be useful to examine laboratory-confirmed asymptomatic cases both as infector and infectee and estimate whether generation time differs, to enable better understanding of disease transmission in such circumstances. However, this was not feasible in our study as the date of exposure was not systematically collected in case-based investigation forms. It would also have been interesting to estimate serial intervals for different demographic, geographic, or exposure (e.g. hospital, non-hospital setting) strata, but this was not feasible due to our limited sample size. Additionally, it would have been interesting to compare serial interval estimates at different phases of the epidemic as suggested by Ali et al. [12], but this was not feasible as it became difficult to identify well-defined chains of transmission once Lebanon entered its community transmission phase in July 2020. Finally, it was not possible for us to conduct a sensitivity analysis with and without scenarios, multi-factorial exposures, and multiple possible primary cases as the cases we excluded were missing the information pertinent for such an analysis.

Our study informed national policy, providing crucial information that fed into contact-tracing measures adopted by Lebanon’s MOPH. For instance, alignment of our results with international findings and recommendations influenced MOPH to recommend contacts be tested within 5–7 days from last exposure to a positive COVID-19 case instead of being tested immediately as was initially advised in April 2020. Thus, we believe our study can serve as an example for other countries to better understand transmission dynamic parameters and implement evidence-informed contact-tracing procedures.

## Conclusion

Identifying the transmission dynamics of SARS-CoV-2 is crucial for evidence-informed decision-making in controlling national COVID-19 epidemics. Our analysis of surveillance and investigation data collected on the first identified transmission chains in Lebanon guided national authorities in the implementation of contact tracing and testing, highlighting the necessity of testing contacts starting the 5th day after exposure rather than immediately upon identification.

Our study methods and findings can serve as a useful example on the importance of conducting contact tracing to understand virus transmission dynamics and tailor response activities accordingly.

## Supplementary Information

**Additional file 1: Figure S1.** Empirical density distribution of the serial interval of the non-truncated data.

## Data Availability

The data supporting study findings are the responsibility of the Epidemiological Surveillance Program of the Lebanese Ministry of Public Health (ESU-MOH). Thus, restrictions apply to the availability of these data, which were used under license for the current study and are not publicly available. Data are, however, available from the authors upon reasonable request and with permission of ESU-MOH.
