# Supplementary material for: Calculating the serial interval of SARS-CoV-2 in Lebanon using 2020 contact-tracing data
Source: BMC Infect Dis. 2021 Oct 11;21:1053. doi: 10.1186/s12879-021-06761-w (PMC8502789; doi:10.1186/s12879-021-06761-w)
Supplement: Supplementary file 1 — Additional file 1: Figure S1. Empirical density distribution of the serial interval of the non-truncated data. [file 12879_2021_6761_MOESM1_ESM.docx]

Additional file

Figure S1. Empirical density distribution of the serial interval of the non-truncated data
